# Cross-cultural adaptation and psychometric evaluation of the Chinese adapted and revised food allergy self-efficacy scale for parents

**DOI:** 10.3389/fpsyg.2026.1750793

**Published:** 2026-02-19

**Authors:** Qi Li, Zhigang Liu, Jiao Li, Min Pan, Yuli Li, Xinxia Chen, Jiao Liu, Hongyue Dai, Yuxin Tang, Lanting Zhao, Ju Wang

**Affiliations:** 1 School of Nursing and Rehabilitation, Cheeloo College of Medicine, Shandong University, Jinan, Shandong, China; 2 Department of Pediatrics, Jinan Maternity and Child Care Hospital Affiliated to Shandong First Medical University, Jinan, Shandong, China; 3 Pediatric Respiratory Department, Shandong Provincial Hospital Affiliated to Shandong First Medical University, Jinan, Shandong, China

**Keywords:** cross-cultural adaptation, CAR-FASE-P, Chinese, food allergy, parental self-efficacy, reliability, validity

## Abstract

**Background:**

Food allergy (FA) in Chinese children is a growing public health concern. Parental FA self-efficacy, characterized by parents’ confidence in managing children’s FA, correlates with parental and children’s wellbeing. However, there is currently no Chinese valid scale for measuring the parental FA self-efficacy. This study culturally adapted and revised the Food Allergy Self-Efficacy Scale for Parents (FASE-P) into Chinese, and examined the psychometric properties of the Chinese adapted and revised FASE-P (CAR-FASE-P).

**Methods:**

Cross-cultural adaptation of the FASE-P was firstly conducted through forward translation, back translation, expert consultation and cognitive interview with parents of FA children. To assess the psychometric properties, a total of 372 parents of FA children were recruited in the outpatient clinic of hospitals in Jinan, Shandong, China, and 30 parents participated in the retest. Item analysis, validity (including content validity, structural validity and criterion-related validity) and reliability (including internal consistency reliability, split-half reliability, test–retest reliability) of the CAR-FASE-P were examined.

**Results:**

After two rounds of expert panel reviews, adequate content validity of the preliminary CAR-FASE-P was confirmed, with the item-level content validity index ranging from 0.800 to 1.000 and the scale-level content validity index of 0.944. Exploratory factor analysis identified 4 factors accounting for 66.074% of the total variance. The modified confirmatory factor analysis model demonstrated that the 5-factor model fitted the data well (χ^2^/df = 1.556, root mean square error of approximation = 0.049, comparative fit index = 0.983, Tucker-Lewis index = 0.981, weighted root mean square residual = 0.911). The total Cronbach’s *α* was 0.912, and the split-half and the test–retest reliability coefficients were 0.815 and 0.798, respectively. The scores of CAR-FASE-P were significantly correlated with General Self-Efficacy Scale scores (*r* = 0.226, *p* < 0.001). The final version of CAR-FASE-P was a five-factor structure composed of 23 items.

**Conclusion:**

Evaluated in a Chinese sample, the CAR-FASE-P demonstrated preliminary evidence for validity and reliability, suggesting its potential utility for assessing parental self-efficacy in food allergy management.

## 1 Introduction

Food allergy (FA) is characterized as an adverse health effect resulting from a specific immune response, which occurs consistently upon exposure to a particular food antigen (Boyce et al., 2010). FA affects an estimated 8% of children worldwide (Spolidoro et al., 2023; Warren et al., 2020), with a steadily rising prevalence over the past several decades (Sicherer and Sampson, 2014; Bartha et al., 2024). Notably, the prevalence of FA among children in China has reached 11.1% (Leung et al., 2024), reflecting a significant public health concern (Feng et al., 2023; Leung et al., 2024). FA can affect multiple organ systems in children, including respiratory, circulatory, digestive systems and skin (Anvari et al., 2019; Sicherer and Sampson, 2010). Beyond physiological effects, FA might increase family economic burdens (Patel et al., 2011), reduce quality of life and cause psychosocial issues like anxiety and depression in both patients and their caregivers (Conway et al., 2024; Polloni and Muraro, 2020; Walkner et al., 2015; Warren et al., 2020). Effective FA management requires coordinated strategies including allergen avoidance and acute response protocols, and also involves a variety of stakeholders including caregivers, schools, the food industry and public health authorities (Sicherer and Sampson, 2018; Boyce et al., 2010; Dupuis et al., 2020; Galletta et al., 2025). However, a recent study showed substantial gaps in parental knowledge and behavioral practices concerning the management of pediatric food allergies (Galletta et al., 2025). For parents, effectively navigating their child’s FA often poses practical parenting challenges, and may consequently adversely impact their confidence in parenting capacities.

In line with Bandura’s Self-Efficacy Theory, an individual’s self-efficacy can exert an influence on their behavioral choices, coping strategies, and other psychological states. Parental self-efficacy, as a domain-specific construct of self-efficacy, is commonly defined as parents’ belief in their capability to effectively fulfill the responsibilities of raising and caring for their children (Jones and Prinz, 2005). Parental FA self-efficacy is characterized by parents’ confidence in their behaviors related to managing their children’s FA (Knibb et al., 2015). Extant research demonstrated that parents’ or other caregivers’ FA self-efficacy correlates positively with their quality of life (Knibb et al., 2016; Pappalardo et al., 2022). Moreover, caregivers’ FA self-efficacy was found to mediate the relationship between perceived allergy severity and caregiving burden (Barber et al., 2025). Additionally, higher parental FA self-efficacy was inversely associated with their anxiety, worry, and posttraumatic stress symptoms (PTSS) (Roberts et al., 2021), and positively linked to health literacy (Ditzler and Greenhawt, 2016). Given these close links to critical outcomes, it is imperative to assess FA self-efficacy in parents of children with FA. However, research on the parental FA self-efficacy in China remains scarce, despite the country’s complex FA landscape characterized by significant geographical and environmental diversity (Leung et al., 2024). A critical barrier to such research is the absence of an appropriate instrument for measuring parental FA self-efficacy in the Chinese context.

The common scales used to evaluate the parental FA self-efficacy globally include the Parenting Sense of Competence scale (PSOC), the Food Allergy Self-Efficacy Questionnaire (FASEQ), the Parental Self-Efficacy Scale for Dietary Management of Children with Food Allergies (PSED-FA) and the Food Allergy Self-Efficacy Scale for Parents (FASE-P). The efficacy subscale of the PSOC has accumulated numerous practical application cases in psychology and parenting research, with a stable measurement framework validated over time. It has thus been effectively applied to research on parents of children with FA (Williams and Hankey, 2016). However, as a general scale for measuring parenting competence, the PSOC does not include items tailored to the specific challenges of raising a child with FA. The FASEQ is a clinically practical tool for assessing caregiver self-efficacy in core FA scenarios but provides limited coverage of management challenges in social or public settings (Ditzler and Greenhawt, 2016), while managing social interactions is a critical domain of FA care (Bollinger et al., 2006). The PSED-FA is a disease-specific scale designed to assess the self-efficacy of parents of children with FA but focuses on self-efficacy related to dietary management (Lee et al., 2025). Compared with them, the FASE-P scale could assess parents’ FA self-efficacy across five dimensions: precaution and prevention of allergic reactions, management of allergic reactions, food allergen identification, information-seeking, and navigation of social activities related to FA. Originally developed for parents of food-allergic children in Britain (Knibb et al., 2015), the scale had been adapted into Turkish (Çalışkan et al., 2024). Studies had reported sound internal consistency for the scale and provided validity evidence via its significant correlations with measures of general self-efficacy, quality of life, and mental health (Knibb et al., 2015; Knibb et al., 2016).

While the psychometric properties of FASE-P had been established in previous studies, those investigations were primarily focused on populations in Western countries (e.g., Britain) and Turkey. Its reliability and validity in other cultural contexts, such as China, remain unverified (Shen et al., 2025). Moreover, there are significant cultural differences between China and the UK, where the scale was originally developed. For instance, one information source listed in the original scale, “G.P. or nurse,” is not applicable within China’s healthcare system. Therefore, formal cultural adaptation and validation are essential steps before the scale FASE-P can be reliably used in the Chinese context.

The present study had two objectives: (1) to translate the FASE-P into Chinese and conduct a cross-cultural adaptation to develop the preliminary culturally adapted and revised FASE-P; (2) to evaluate the reliability and validity of the preliminary scale in the target population and further refine it to form the final version of CAR-FASE-P. Our study may provide a useful tool for assessing the parental FA self-efficacy in China, thus paving the way for developing effective interventions to enhance it.

## 2 Methods

### 2.1 Cross-cultural adaptation

We secured authorization from the original author of the FASE-P via email prior to adaptation. Cross-cultural adaptation was conducted with reference to rigorous methodological guidelines (Beaton et al., 2000). The detailed procedures were described as follows.

#### 2.1.1 Forward translation and back translation

Forward translation: Two bilingual translators independently conducted the forward translation of the FASE-P from English to Chinese. One translator was a clinical physician specializing in childhood FA, and the other was a nursing faculty member who was blinded to the specific concepts being measured. Both translators held PhD degrees, had overseas academic experience, and were proficient in both Chinese and English. Subsequently, the research team reviewed both translated versions alongside the original English version. Any discrepancies were discussed until a consensus was reached, resulting in a synthesized Chinese version, namely the CAR-FASE-P (version 1).

Back translation: The CAR-FASE-P (version 1) was back-translated into English by two bilingual translators. Both translators had no prior knowledge of the original scale, had no medical background, and held bachelor’s or master’s degrees in English, along with the Test for English Majors-Band 8 certificate (the highest-level national qualification test for English majors in Chinese universities). The two back-translated versions were compared and reconciled against each other by two translators and the finalized English back-translated version was agreed upon through discussion.

We then sent the finalized English back-translated version of the scale to the original author of FASE-P via email for review, requesting verification of the equivalence between all translated content and the original questionnaire across semantic, idiomatic, experiential and conceptual dimensions. The author confirmed that the English back-translated version of the CAR-FASE-P (version 1) was meaningfully consistent with the original scale, with no revisions required.

#### 2.1.2 Expert consultation

The multidisciplinary expert panel comprised the following members: two specialists in psychological nursing, two senior pediatricians with approximately three decades of experience in the field of FA treatment and holding senior professional titles, and one nurse supervisor with 14 years of clinical experience in care of FA children and their family. These experts provided feedback on the CAR-FASE-P (version 1), focusing on title accuracy, scoring methodology, dimensional and item translation, relevance weighting and linguistic appropriateness using a 5-point Likert scale. A total of two rounds of expert consultation were conducted. Their recommendations were systematically integrated to develop the CAR-FASE-P (version 2) for cognitive interviewing with parents of FA children.

In the CAR-FASE-P (version 2), modifications were made to some items, and new items were added. Within the dimension of *Allergic reaction*, based on expert advice, the item “Treat my child if they had an allergic reaction” was split into two items “Treat my child with appropriate non-pharmacological measures if they had an allergic reaction, such as immediately discontinuing the consumption of the food that triggered the allergy” and “Treat my child with appropriate pharmacological measures if they had an allergic reaction, such as taking cetirizine as prescribed by the physician”. In addition, as suggested by experts, the item “Observe and clarify the progression of my child’s allergic reaction” was added. Within the dimension of *Food allergy identification*, we added brief glosses for professional terms that might be challenging for participants to understand. For example, the item “Identify possible food cross-contamination” was revised to “Identify potential food cross-contamination (where previously safe foods become contaminated with allergenic substances)”. Within the dimension of *seeking information about food allergy*, in light of the varied nature of information sources, the items “Books” and “Other parents of children with FA” were added. Within the dimension of *managing social activities around food allergy*, the items “Dine at a restaurant,” “Vacation within the country” and “Vacation abroad” were all amended to include the clarification “without triggering FA in children” to specify the purpose of social management.

#### 2.1.3 Cognitive interview

Using a convenience sampling method, we recruited 10 parents of children with FA for cognitive interviews from a maternity and child care hospital in Jinan, Shandong, China. We explained the purpose and significance of the study to participants and obtained their informed consent. When data saturation was reached, the sample size was deemed sufficient. Semi-structured interviews were conducted with each interviewee individually using an interview guide, aiming to understand their comprehension of each item, whether there were ambiguities in the expression of scale items and whether any items needed to be added or removed. Notes were taken during the interview process. Subsequently, the data were analyzed by two researchers through content analysis. Aside from some ambiguities in expression, participants reported that the percentage scoring system created ambiguity, as the distinctions between scores were so fine that they struggled to assign themselves an appropriate one. This opinion aligned with that of the experts, who also cautioned that the percentage scoring system could lead to ceiling or floor effects and thus recommended changing it to a Likert scale. Therefore, informed by the scoring method of the General Self-Efficacy Scale (GSES), we adopted a 4-point Likert scale for the scale. After completing the cognitive interviews and organizing discussions on the collected feedback, the CAR-FASE-P (version 3) was developed.

### 2.2 Psychometric testing

#### 2.2.1 Study design and participants

The cross-sectional study recruited eligible parents of food-allergic children via a convenience sampling in the outpatient clinics of a maternity and child care hospital and a general hospital in Jinan, Shandong, China, from February 2025 to May 2025. The inclusion criteria for participants in this study were as follows: being aged ≥18 years; being a father or mother of at least one child with a physician-confirmed FA; having the child’s diagnosis meet the criteria for FA in the *Evidence-Based Guidelines for Food Allergy in Chinese Children*, specifically through a positive oral food challenge test, or a convincing clinical history of FA combined with a positive specific IgE or skin prick test (Zhou et al., 2022); being the primary caregiver of the child; being capable of normal understanding and communication; and providing informed consent and voluntarily participating in this study. The parents were excluded if their child with FA or they themselves suffered from severe physical diseases such as malignant tumors, or had intellectual or mental disorders. For Exploratory Factor Analysis (EFA), the sample size should be 5–10 times the number of scale items (Costello and Osborne, 2005). This study further accounts for an extra 10% of invalid samples, resulting in a minimum total sample size of 138. For Confirmatory Factor Analysis (CFA), a minimum sample size of 200 is required (Westland, 2010). Finally, we totally recruited 372 participants. In this study, the sample was split into two segments based on the sequence of questionnaire collection. For factor analysis, 138 of them was assigned to the EFA group and 234 to the CFA group. We compared the key demographic characteristics and self-efficacy scores of these two subgroups and found no statistically significant differences. Detailed comparison results were presented in Supplementary Table S1. Thirty parents of children with FA were conveniently selected from the whole sample for a repeated assessment two to four weeks after the first test to calculate the test–retest reliability of the scale.

This study was approved by the Ethics Committee of the School of Nursing and Rehabilitation, Shandong University. All participants provided consent to participate.

#### 2.2.2 Measures

The questionnaires were distributed at the outpatient clinics and were filled out by the parents after their medical appointment. The questionnaire was composed of the demographic questionnaire, the CAR-FASE-P (version 3), and the GSES.

1) Demographic questionnaire

The questionnaire collected the participants’ demographic data including their age (year), relationship to the child (father, mother), educational level (primary school, middle school, high school, college graduate or bachelor’s, higher than bachelor’s), monthly household per capita income (<2,000, 2,000–5,000, >5,000–10,000, >10,000–15,000, >15,000) and marital status (single, married, divorced), along with child’s age (month), child’s gender (male, female), family residential location (urban area, suburban area, county town), family history of allergic diseases (yes, no), FA child’s food allergen and FA-related symptoms (gastrointestinal symptoms, respiratory symptoms, skin symptoms, other symptoms).

2) The CAR-FASE-P (version 3)

The CAR-FASE-P (version 3) was developed to measure the parental FA self-efficacy. This scale consisted of 25 items, with an item score range of 1 to 4 points. Scores were defined as follows: 1 indicated “cannot do it at all,” 2 indicated “moderately uncertain to be able to do it,” 3 indicated “moderately certain to be able to do it,” and 4 indicated “highly certain can do it.” The total mean score of the scale is calculated as the average of the scores of all items, and a higher score indicates better self-efficacy among the parents of children with FA. The items of CAR-FASE-P (version 3) in English were shown in Supplementary Table S2.

3) General Self-Efficacy Scale

GSES was originally developed by Ralf Schwarzer, a German clinical and health psychologist (Villegas Barahona et al., 2018). The Chinese version of the General Self-Efficacy Scale was translated, adapted, and revised by Wang et al. (2001). This scale consists of 10 items, which are scored using a 4-point Likert scale. The total score of the scale is calculated by summing the scores of all items and then dividing by 10. With 2.5 as the reference score, values below this threshold indicate a low level of self-efficacy, whereas scores above it correspond to higher levels of self-efficacy. This scale was used as a criterion tool to assess the criterion-related validity of the CAR-FASE-P.

#### 2.2.3 Data analysis

##### 2.2.3.1 Item analysis

Item analysis was conducted through the critical ratio method, the correlation coefficient method, and the Cronbach’s *α* coefficient method. The top 27% of total scale scores constituted the high-score group, while the lowest 27% formed the low-score group. Independent sample *t*-tests were conducted on the scores of each item between the two groups. A critical ratio (CR) > 3.00 indicated good discriminative validity for the items (Peng et al., 2024). Items were considered to have reasonable homogeneity if the correlation coefficient between each item score and the total scale score was ≥0.3 (Galanis et al., 2023). A lack of increase in the scale’s Cronbach’s *α* coefficient after removing a given item, as indicated by Cronbach’s α analysis, supports the retention of that item (Peng et al., 2024).

##### 2.2.3.2 Validity analysis

Validity analysis refers to the degree to which a measurement instrument accurately assesses the intended construct (Messick, 1995). In this study, validity was evaluated from three aspects: content validity, structural validity and criterion-related validity. Five experts were invited to evaluate the content validity of the CAR-FASE-P. The evaluation process utilized the item-content validity index (I-CVI) and the scale-content validity index (S-CVI). A good content validity of the scale was indicated when I-CVI > 0.780 and S-CVI > 0.900 (Shi et al., 2012).

EFA and CFA were used to evaluate the structural validity. EFA with principal component analysis and varimax orthogonal rotation was appropriate when the Bartlett’s test of sphericity was significant (*p* < 0.001) and the Kaiser-Meyer-Olkin (KMO) measure exceeded 0.8 (Hair et al., 2013). For CFA, the weighted least squares mean and variance (WLSMV) adjusted estimation was applied. WLSMV was recommended for application in scenarios where the number of rating categories is fewer than five (Beauducel and Herzberg, 2006). Model fitness was considered acceptable when the goodness-of-fit indices met the following criteria: chi-square to degrees of freedom ratio (χ^2^/df) < 3.00 (Iacobucci, 2010), root mean square error of approximation (RMSEA) ≤ 0.06 (Hu and Bentler, 1999), comparative fit index (CFI) and Tucker-Lewis index (TLI) ≥ 0.97 (Schermelleh-Engel et al., 2003), and weighted root mean square residual (WRMR) ≤ 0.95 (Yu, 2002).

Criterion-related validity was evaluated by examining the Pearson correlation between the CAR-FASE-P and the GSES. The strength of the correlation was defined as follows: low (*r* ≤ 0.35), moderate (*r* = 0.36–0.67), or high (*r* = 0.68–1.0) (Taylor, 1990).

##### 2.2.3.3 Reliability analysis

Reliability analysis estimates the consistency of scale measurements. Internal consistency reliability was evaluated using Cronbach’s *α* coefficient, with ≥0.70 generally considered indicative of scale usability (Zhong et al., 2024). Split-half reliability was estimated by calculating the correlation between the two halves created using the odd-even method, and then applying the Spearman-Brown prophecy formula for correction; a coefficient >0.80 indicates good utility of the questionnaire (Chen et al., 2024). Test–retest reliability was evaluated by calculating the Pearson correlation coefficient of questionnaire scores for a subset of 30 parents, with >0.70 indicating high consistency between two measurements and good scale stability (Li et al., 2025).

### 2.3 Sensitivity analysis

The median age of the child with FA was 8 months (IQR: 4–16 months), so most of the parents had not yet experienced scenarios such as school enrollment described by some scale items, which might inflate ceiling effects or distort the factor structure. Therefore, we conducted a sensitivity analysis by performing CFA across child age subgroups to assess the robustness of the factor structure. Since the subgroup of parents with FA children aged 1 year or older had fewer than 200 participants (below the minimum sample size requirement for CFA), we performed CFA exclusively in the subgroup of children under 1 year old (*n* = 243). We also compared CAR-FASE-P scores between parents of children across age subgroups (<1 year vs. ≥1 year). In addition, considering that sequence-based allocation might introduce systematic differences, we used a random number table applied to each participant’s identification number to select 150 participants from the entire sample for EFA, while the remaining 222 participants were used for CFA.

## 3 Results

### 3.1 Descriptive statistics

A total of 372 parents of children with FA were recruited for the questionnaire survey. The demographic data of the parents of children with FA and the demographic data of these children were, respectively, presented in Tables 1, 2. The average age of the parents of children with FA participating in the survey was 31.518 ± 4.707 years old, of which 79.6% were mothers. Seventy-five percent of parents held a college or bachelor’s degree, more than half reported a monthly household per capita income ranging between RMB 5,000 and 10,000 yuan, and nearly all were in a married status. The median age of the children was 8 months (IQR, 4–16 month), including 219 boys and 153 girls. 77.7% of the children lived in urban areas. The vast majority of children came from families with only one child, and more than half of the children had a family history of allergic diseases. Milk was the most common allergen, accounting for the vast majority of cases, while egg allergies were reported by nearly half of the children.

**Table 1:** Demographic characteristics of parents (*n* = 372).

Variables	Number (%)/Mean ± SD
Parents	
Age (Year)	31.518 ± 4.707
Relationship to the child	
Father	76 (20.4)
Mother	296 (79.6)
Educational level	
Primary school	2 (0.5)
Middle school	14 (3.8)
High school	38 (10.2)
College graduate or bachelor’s	279 (75)
Higher than bachelor’s	39 (10.5)
Monthly household per capita income (yuan)	
<2,000	8 (2.2)
2,000–5,000	45 (12.1)
>5,000–10,000	192 (51.6)
>10,000–15,000	96 (25.8)
>15,000	31 (8.3)
Marital status	
Single	1 (0.3)
Married	368 (98.9)
Divorced	3 (0.8)

**Table 2:** Demographic characteristics of children with FA (*n* = 372).

Variables	Number (%)/Median (Q1, Q3)
Age (Month)	8.00 (4.00, 16.00)
Gender	
Male	219 (58.9)
Female	153 (41.1)
Family residence location	
Urban area	289 (77.7)
Suburban area	28 (7.5)
County town	44 (11.8)
Rural area	11 (3.0)
Family history of allergic diseases	
Yes	201 (54.0)
No	171 (46.0)
Food allergen reported	
Milk	322 (86.6)
Egg	136 (36.6)
Seafood	58 (15.6)
Wheat	36 (9.7)
Soy products	30 (8.1)
Peanut	12 (3.2)
Others	9 (2.4)
Symptom of FA	
Gastrointestinal symptoms	219 (58.9)
Respiratory symptoms	157 (42.2)
Skin symptoms	148 (39.8)
Other symptoms	38 (10.2)

### 3.2 Item analysis

As show in Table 3, the results of item analysis showed that the critical ratio of all items ranged between 5.250 and 15.566, indicating that all items on the scale had good differentiation. The correlation coefficients between each item and the total score were between 0.333 and 0.723 (all *p* < 0.01), suggesting good discrimination. After deleting one item at a time, the Cronbach’s *α* values for the scale ranged from 0.915 to 0.921, all of which were lower than or equal to the Cronbach’s *α* coefficient of the total scale including 25 items. The specific item content corresponding to the item numbers is presented in Supplementary Table S2.

**Table 3:** Item analysis and descriptive information for CAR-FASE-P (version 3).

Item number	Critical ratio	Correlation coefficient between item and total score	Cronbach’s *α* if item deleted	Mean ± SD
A1	7.030	0.484	0.919	3.81 ± 0.56
A2	7.981	0.449	0.919	3.75 ± 0.64
A3	7.011	0.454	0.919	3.78 ± 0.60
A4	9.067	0.470	0.919	3.65 ± 0.75
A5	5.250	0.333	0.921	3.87 ± 0.48
A6	8.305	0.545	0.918	3.37 ± 0.65
B1	8.429	0.530	0.918	3.76 ± 0.58
B2	5.368	0.559	0.918	3.87 ± 0.46
B3	5.963	0.512	0.918	3.84 ± 0.52
B4	5.974	0.615	0.918	3.87 ± 0.44
C1	6.195	0.545	0.918	3.85 ± 0.50
C2	9.133	0.572	0.917	3.76 ± 0.58
C3	5.899	0.565	0.918	3.86 ± 0.49
D1	9.410	0.415	0.921	3.60 ± 0.87
D2	5.763	0.470	0.919	3.88 ± 0.44
D3	15.566	0.490	0.921	3.28 ± 1.07
D4	10.324	0.541	0.918	3.61 ± 0.81
D5	12.755	0.632	0.916	3.49 ± 0.91
D6	11.875	0.596	0.917	3.58 ± 0.79
E1	6.999	0.700	0.916	3.82 ± 0.54
E2	7.981	0.686	0.915	3.77 ± 0.62
E3	7.457	0.716	0.915	3.80 ± 0.56
E4	7.960	0.723	0.915	3.78 ± 0.59
E5	9.347	0.689	0.915	3.66 ± 0.77
E6	10.455	0.671	0.915	3.57 ± 0.88
Cut-off values	≥3	≥0.3	≤0.921^a^	

a The total Cronbach’s *α* coefficient of the total scale was 0.921.

### 3.3 Validity analysis

#### 3.3.1 Content validity analysis

Five experts in related fields were invited to review the content validity of the CAR-FASE-P. In the first round of expert consultation, the I-CVI ranged from 0.600 to 1.000, and the S-CVI was 0.905. In the second round of expert consultation, the I-CVI ranged from 0.800 to 1.000, and the S-CVI was 0.944.

#### 3.3.2 EFA

The results of EFA showed that the KMO value was 0.812 (χ^2^ = 2,481.815, *p* < 0.001), indicating that the sample was suitable for factor analysis. After the initial EFA, items A6 and E1 were deleted, as they exhibited cross-factor loadings and their factor loading differences were less than 0.20. The results of the initial EFA for CAR-FASE-P (version 3) was presented in Supplementary Table S3. The second EFA was then conducted, and the final results are presented in Table 4. A total of 4 common factors with eigenvalues >1 were extracted, and the cumulative variance contribution rate was 66.074%. All items showed factor loadings exceeding 0.40. Based on the results of EFA and combined with the content of the items, the research team decided to classify items A4 and D2 into dimension *managing social activities around food allergy* and dimension *seeking information about food allergy, respectively,* after discussion. To this point, we had developed the CAR-FASE-P (version 4).

**Table 4:** Factor loadings of second EFA for CAR-FASE-P.

Item Number	Factor 1	Factor 2	Factor 3	Factor 4
A1	0.097	0.232	0.039	**0.840**
A2	0.188	0.207	0.176	**0.763**
A3	0.124	0.192	0.028	**0.706**
A4	−0.058	**0.542**	0.273	0.390
A5	0.148	0.086	0.002	**0.679**
B1	**0.744**	0.080	0.320	0.115
B2	**0.819**	0.055	0.209	0.157
B3	**0.726**	0.081	−0.048	0.105
B4	**0.822**	0.259	0.077	0.113
C1	**0.773**	0.034	0.129	0.218
C2	**0.589**	0.172	0.109	0.160
C3	**0.768**	0.162	0.120	−0.045
D1	−0.008	0.208	**0.662**	0.146
D2	**0.646**	0.438	0.152	0.002
D3	0.176	0.061	**0.786**	−0.060
D4	0.213	0.170	**0.803**	−0.007
D5	0.088	0.299	**0.792**	0.247
D6	0.399	0.165	**0.735**	0.029
E2	0.115	**0.715**	0.139	0.263
E3	0.181	**0.771**	0.216	0.279
E4	0.319	**0.810**	0.178	0.074
E5	0.214	**0.824**	0.149	0.192
E6	0.179	**0.838**	0.157	0.089
Cumulative variance contribution rate (%)	37.154	49.454	59.158	66.074

The bold values indicate the highest factor loading for each item.

#### 3.3.3 CFA

**Figure 1:**
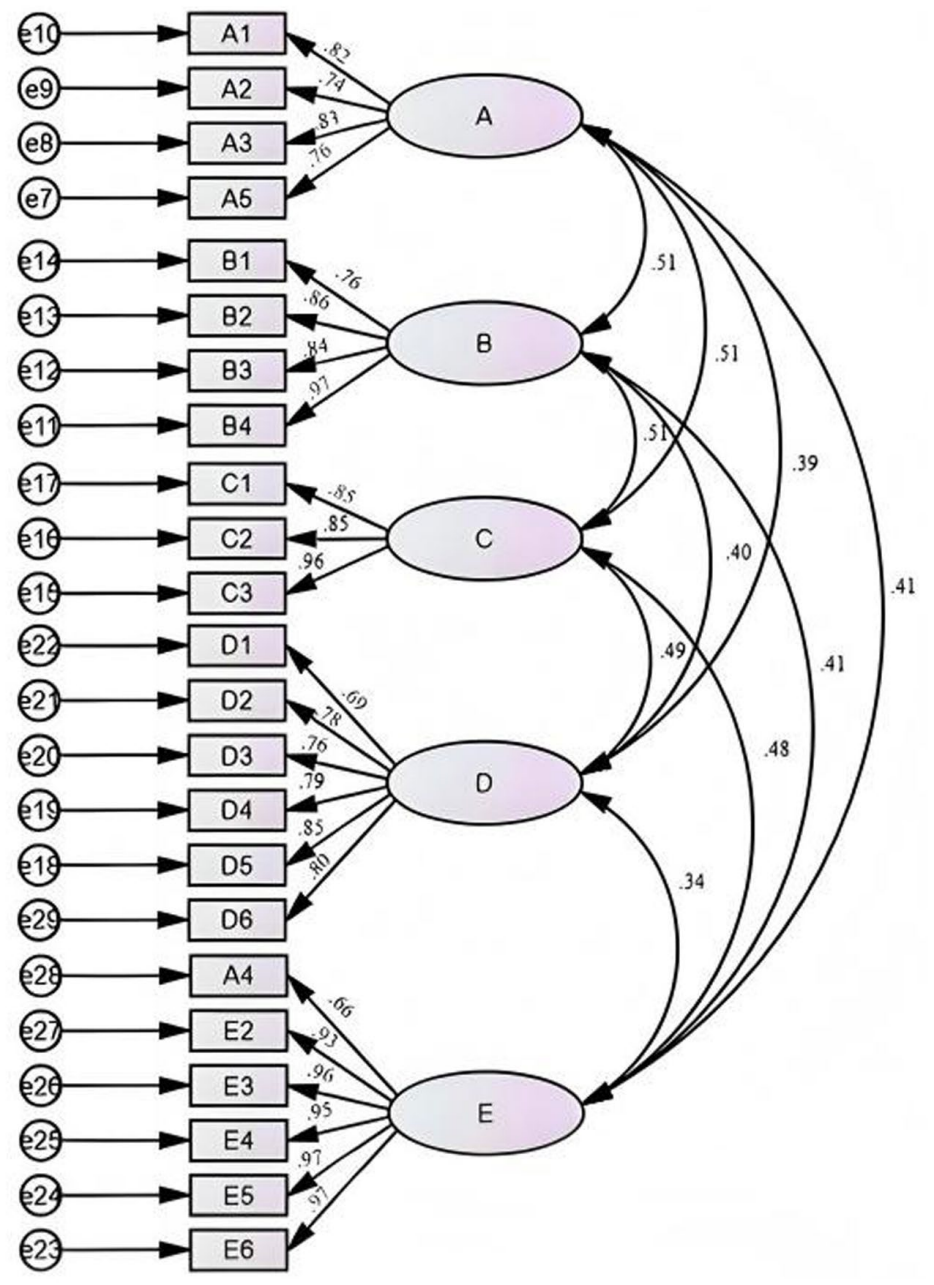
Standardized five-factor model of the CAR-FASE-P.

We firstly conducted CFA on the four-factor model derived from EFA and the values of χ^2^/df, RMSEA, CFI, TLI, and WRMR were 1.599, 0.051, 0.982, 0.979, and 0.957, respectively, indicating a good model fit. In the four-factor model, the items B1, B2, B3, B4 (addressing allergic reactions) and items C1, C2, C3 (addressing food allergy identification) were all loaded onto Factor 1. However, “allergic reaction” and “food allergy identification” have been recognized as two distinct aspects of food allergy management in previous studies (Boyce et al., 2010; Burks et al., 2012). “Allergic reactions” typically focus on the strategies about emergency intervention and symptom control following the onset of an allergic event; while “food allergy identification” aims to pinpoint and trace allergy triggers, targeting their localization before or during an allergic reaction. Based on that, we considered splitting Factor 1 into two factors: items B1, B2, B3, and B4 pertained to “allergic reaction,” and items C1, C2, C3 pertained to “food allergy identification,” thus forming a five-factor model which was largely consistent with the original conceptual dimensions. As presented in Table 5, the model indices of the five-factor model (χ^2^/df = 1.556, RMSEA = 0.049, CFI = 0.983, TLI = 0.981, WRMR = 0.911) suggested a better fit to the data than the four-factor model. We thereby adopted the five-factor scale with 23 items (version 5) in the following analyses. Figure 1 shows the five-factor model constructed by CFA.

**Table 5:** Model fit index comparison table for CFA models for CAR-FASE-P.

Model / cut-off value	χ^2^/df	RMSEA (95% CI)	CFI	TLI	WRMR
Four-factor model	1.599	0.051 (0.041, 0.060)	0.982	0.979	0.957
Five-factor model	1.556	0.049 (0.038, 0.059)	0.983	0.981	0.911
Cut-off values	<3	≤0.06	≥0.97	≥0.97	≤0.95

#### 3.3.4 Criterion-related validity analysis

This study employed the GSES to evaluate the criterion-related validity of the CAR-FASE-P (version 5). The scores of the two scales were significantly correlated (*p* < 0.001) although the correlation strength was low (*r* = 0.226, data not shown).

### 3.4 Reliability analysis

The results of the reliability analysis for the CAR-FASE-P (version 5) were presented in Table 6. The Cronbach’s *α* coefficients of the CAR-FASE-P and its subdimensions ranged between 0.728 and 0.912, and the split-half reliability of the total scale was 0.815 and the test–retest reliability was 0.798. At this point, we finalized the CAR-FASE-P with 23 items across 5 factors. The generation process for the different versions of the CAR-FASE-P was presented in the Supplementary Figure S1. The final 23-item instrument in Engilsh was presented in the Supplementary Table S4. Among all 372 participants enrolled in this study, the total mean score of the CAR-FASE-P was 3.72 (SD = 0.39).

**Table 6:** Reliability analysis for CAR-FASE-P (version 5).

Scale and dimension	Cronbach’s *α*	Split-half reliability	Test–retest reliability
The CAR-FASE-P	0.912	0.815	0.798
Precaution and prevention of an allergic reaction	0.728		
Allergic reaction	0.824		
Food allergy identification	0.751		
Seeking information about food allergy	0.806		
Managing social activities around food allergy	0.887		

### 3.5 Sensitivity analysis results

Sensitivity analysis demonstrated good model fit indices for CFA among parents of FA children under 1 year old, with detailed results presented in the Supplementary Table S5. We compared CAR-FASE-P scores between parents of children <1 year (Mean = 3.74, SD = 0.38) and ≥1 year (Mean = 3.69, SD = 0.40) and found no statistically significant difference (*t* = 1.218, *p* = 0.224). Furthermore, the EFA results based on simple random sampling were largely consistent with those based on sequential sampling. In the first EFA, consistent with the primary findings, the items A6 and E1 exhibited cross-loadings with a difference of less than 0.2 (please see Supplementary Table S6). Therefore, these two items were removed, and a second EFA was conducted on the remaining items. The results of the second EFA were all consistent with the primary findings, except that the factor loading of item A5 did not exceed 0.4; however, its highest loading was still observed on Factor 4. Meanwhile, given the importance of this item to assessing the parental FA self-efficacy, it was retained in dimension *managing social activities around food allergy* of the scale. Then we performed a CFA, and the model fit indices reached satisfactory levels (χ^2^/df = 1.103, RMSEA = 0.022, CFI = 0.999, TLI = 0.999, WRMR = 0.869). Detailed results of the second EFA and CFA based on random sampling are presented in Supplementary Tables S7, S8.

## 4 Discussion

In this study, we performed translation, cultural adaptation, and psychometric validation of the FASE-P. The results demonstrated that the finalized version of CAR-FASE-P, consisting of 23 items and 5 dimensions, was a scale with good reliability and initial construct validity, which was capable of evaluating the self-efficacy level of parents of Chinese children with FA.

During the process of scale development and optimization, the scientific nature of the scoring method directly affected the accuracy of data and the reliability of research conclusions. As self-efficacy itself is a subjective psychological perception (Bandura and Locke, 2003), it is difficult to define it with precise percentage counts. Excessively fine-grained rating scales are inconsistent with the cognitive habits of respondents and increases their scoring burden. Ultimately, based on a synthesis of iterative expert reviews, insights from cognitive interviews, and the scoring methodology of the GSES, the research team ultimately determined to adopt a 4-point Likert scale for CAR-FASE-P to align with established measurement practices. Moreover, we modified the content of some items during the sinicization process. To enhance understanding, researchers added explanatory content to some items during the translation and cultural adaptation of the FASE-P. For example, “food cross-contamination” was a professional term that participants might not fully comprehend. Therefore, with reference to the C-FAQL-PB scale (Chen, 2022), a clarification on this term “where previously safe foods become contaminated with allergenic substances” was included. Additionally, in consideration of the fact that some parents in China acquire parenting knowledge through books (He and Chen, 2025) and that there is peer support among parents of children with FA (Chooniedass et al., 2020), two items were additionally added regarding the ways to seek FA information—“books” and “parents of other children with FA”—to account for the diversity of information sources among Chinese parents. Based on the guidelines for managing FA in Chinese children (Zhou et al., 2022), FA treatment measures were categorized into pharmacological and non-pharmacological treatments according to the severity of allergic reactions. Therefore, the item about treatment measures was further divided into pharmacotherapy and non-pharmacotherapy in the CAR-FASE-P.

Due to our revisions mentioned above, direct score comparisons with the original and Turkish versions of FASE-P should be made with caution. However, these adaptations were necessary to establish functional and conceptual equivalence in the Chinese context, thereby enhancing the tool’s validity for local use. The comparability now lies more in the instrument’s ability to predict relevant outcomes or correlate with key constructs, rather than in literal score equivalence. We frame this revised version as a crucial step for meaningful cross-cultural research, not a limitation. Considering both the interpretation of the 4-point Likert scale and the established GSES cutoff of 2.5 for distinguishing between low and high levels of self-efficacy, we propose that when using the CAR-FASE-P, a total mean score ≥2.5 indicates a high level of parental FA self-efficacy, whereas a score <2.5 indicates a low level.

The item analysis of the CAR-FASE-P showed that the critical ratio and the correlation coefficient between each item and the total score were all greater than the cutoff values. This indicates that the scale effectively distinguishes between items (Raykov and Marcoulides, 2016), and each item accurately assesses the self-efficacy of different parents of children with FA. Deletion of any item did not increase the Cronbach’s *α* value of the remaining items. Therefore, no items were removed in this step.

Validity pertains to how accurately and appropriately a questionnaire measures what it is intended to (Li et al., 2025). Content validity assesses the correlation between the items of a scale and the underlying dimensions of the scale. In this study, the I-CVI and S-CVI from the second round of expert consultation both met the required standards, indicating that the scale has strong content validity. The structural validity analysis includes EFA and CFA. The EFA generated a model with 23 items and 4 factors. The cumulative variance explained by the scale reached 66.074%, which exceeded the standard threshold of 50% (Fabrigar et al., 1999). Additionally, all item loadings on their respective factors range from 0.542 to 0.840, surpassing the minimum threshold of 0.40 (Mokkink et al., 2010). The CFA showed that the fitting indices of the 23-item 5-factor model (χ^2^/df = 1.556, RMSEA = 0.049, CFI = 0.983, TLI = 0.981, WRMR = 0.911) were better than those of the 23-item 4-factor model (χ^2^/df = 1.599, RMSEA = 0.051, CFI = 0.982, TLI = 0.979, WRMR = 0.957). Finally, the CAR-FASE-P was determined to be a 23-item, 5-dimensional scale. These results collectively indicate initial construct validity for the scale’s structure.

The reliability of the CAR-FASE-P was evaluated using Cronbach’s *α* coefficient, test–retest reliability and split-half reliability. The total Cronbach’s *α* of the CAR-FASE-P was estimated to be 0.912, which is higher than that of the original version (Cronbach’s *α* = 0.88) (Knibb et al., 2015) and Turkish version of the FASE-P (Cronbach’s *α* = 0.89) (Çalışkan et al., 2024). This indicates that the Chinese culturally adapted and revised version of the scale has good internal consistency. In addition, the split-half reliability of the CAR-FASE-P was 0.815, which further confirms its good reliability (Ma et al., 2022). The test–retest reliability was 0.798, indicating excellent temporal stability (Frost et al., 2007). These indicators all demonstrate that the CAR-FASE-P has good reliability. Among our subjects, the correlation between CAR-FASE-P score and GSES score was low yet significant, indicating weak criterion-related validity. This may be attributed to the conceptual divergence and inherent linkage between the two measures: the GSES assesses an individuals’ comprehensive confidence levels across a wide range of life domains, whereas the CAR-FASE-P captures a domain-specific construct of self-efficacy, specifically focusing on parents’ confidence in their behaviors related to managing their children’s FA. Our results indicate that the GSES can serve as a proxy measurement tool of parental FA self-efficacy. Therefore, we suggest future studies adopt domain-specific external criteria to fully establish the convergent and discriminant validity of the scale.

In the total sample, the mean total score of the CAR-FASE-P was 3.72 (SD = 0.39). This indicates that the parents of children with FA had a relatively high level of self-efficacy. This may be because the subjects recruited in the study were all from the outpatient departments of two Grade A tertiary hospitals in Shandong Province—these children themselves had relatively mild symptoms and their parents might have a relatively high level of awareness regarding their children’s FA. However, parents of children with more severe symptoms who were hospitalized, as well as those with lower educational attainment and cognitive levels were not included. Social and environmental factors strongly influence health-related issues (Tankampuan et al., 2025). Several previous studies on the self-efficacy of parents of children with FA have also shown that their self-efficacy level were indeed in a relatively high level (Knibb et al., 2016; Pappalardo et al., 2022). Research on the parental FA self-efficacy is still at the initial stage in China (Chen, 2022), underscoring the need for further investigation.

Our study has several strengths. Firstly, our study provides an assessment tool for evaluating parental FA self-efficacy in China. Secondly, the CAR-FASE-P facilitates the implementation of interventions aimed at boosting parental FA self-efficacy, enables evaluation of intervention effectiveness and thus allows timely adjustment of strategies. Our study has a limitation of the ceiling effect, this may be associated with the potential bias caused by our convenience sampling. The study sample was mostly derived from urban-dwelling and highly educated mothers, whose children were treated at tertiary hospitals, with mild illness and access to relatively favorable medical resources and support. These characteristics resulted in a generally high level of self-efficacy among the sample, as has been indicated by previous research (Knibb et al., 2016). In addition, this study primarily involved parents of infant FA children who lacked direct experience with scale items such as school safety or vacation planning. Despite instructions at the beginning of CAR-FASE-P clarifying that items assessed confidence even for unexperienced situations, this inexperience may have led to inflated self-efficacy ratings due to difficulties in anticipating actual challenges. The potential bias might artificially inflate scale scores, leading to inherently higher self-efficacy ratings, as well as compromising the scale’s factor structure. A homogeneous sample can disproportionately amplify item loadings that reflect the specific characteristics of that group, thereby limiting the representativeness and generalizability of the extracted factor dimensions. Future validation studies are therefore warranted to be conducted in more diverse contexts, including primary/secondary medical institutions, rural populations and families with lower levels of education.

## 5 Conclusion

In general, this study demonstrated that the 23-item, 5-dimensional CAR-FASE-P is a reliable tool and demonstrates good initial construct validity for assessing the self-efficacy of parents of children with FA in the Chinese context. The adaptation and validation of this scale can fill the gap in the lack of standardized assessment tools for parental FA self-efficay in China and provide support for subsequent studies. Nevertheless, the application of the CAR-FASE-P requires more extensive studies involving larger samples across a wider range of environments and regions.

## Data Availability

The raw data supporting the conclusions of this article will be made available by the authors, without undue reservation.
