# Supplementary material for: Cross-cultural adaptation and psychometric evaluation of the Chinese adapted and revised food allergy self-efficacy scale for parents
Source: Front Psychol. 2026 Feb 19;17:1750793. doi: 10.3389/fpsyg.2026.1750793 (PMC12960144; doi:10.3389/fpsyg.2026.1750793)
Supplement: Supplementary file 1 [file Supplementary_file_1.docx]

**Supplementary Material**

**Supplementary Table S1 Comparison of data of parents and children between EFA group (n=138) and CFA group (n=234).**

| Characteristics | EFA group (n=138) | CFA group (n=234) | χ^2^/ t | P value |
| --- | --- | --- | --- | --- |
|  | n (%)/Mean±SD | n (%)/Mean±SD |  |  |
| Parents |  |  |  |  |
| Age (Year) | 31.459±0.426 | 31.218±0.326 | 0.320 | 0.749 |
| Relationship to the child |  |  | 0.101 | 0.751 |
| Father | 27 (19.57) | 49 (20.94) |  |  |
| Mother | 111 (80.43) | 185 (79.06) |  |  |
| Educational level |  |  | 5.734 | 0.125 |
| Primary school | 0 (0) | 2 (0.85) |  |  |
| Middle school | 9 (6.52) | 5 (2.14) |  |  |
| High school | 14 (10.14) | 24 (10.26) |  |  |
| College graduate, bachelor’s degree, or higher | 115 (83.34) | 203 (86.75) |  |  |
| Monthly household per capita income (yuan) |  |  | 3.657 | 0.600 |
| <2000 | 4 (2.90) | 4 (1.71) |  |  |
| 2000~5000 | 16 (11.59) | 29 (12.39) |  |  |
| **>**5000~10000 | 70 (50.72) | 126 (53.85) |  |  |
| **>**10000~15000 | 33 (23.91) | 57 (24.36) |  |  |
| **>**15000 | 15 (10.87) | 18 (7.69) |  |  |
| Marital status |  |  | 2.847 | 0.241 |
| Single | 1 (0.72) | 0 (0) |  |  |
| Married | 135 (97.83) | 233 (99.57) |  |  |
| Divorced | 2 (1.45) | 1 (0.43) |  |  |
| Score of C-FASE-P | 3.913 (3.712, 4.000) | 3.913 (3.717, 4.000) | -0.400 | 0.689 |
| Children |  |  |  |  |
| Age (Month) | 14.62±1.857 | 15.57±1.348 | -0.570 | 0.569 |
| Family residence location |  |  | 5.902 | 0.116 |
| Urban area | 111 (80.43) | 184 (78.63) |  |  |
| Suburban area | 5 (3.62) | 21 (8.97) |  |  |
| County town | 19 (13.77) | 21 (8.97) |  |  |
| Rural area | 3 (2.17) | 8 (3.42) |  |  |
| Family history of allergic diseases |  |  | 1.004 | 0.316 |
| Yes | 77 (55.80) | 118 (50.43) |  |  |
| No | 61 (44.20) | 116 (49.57) |  |  |

**Supplementary Table S2 Items of the Chinese version of the Food Allergy Self-Efficacy Scale for Parents (CAR-FASE-P) (version 3) in English.**

| Factor | Item Number | Item |
| --- | --- | --- |
| A: Precaution and prevention of an allergic reaction | A1 | Be well-prepared before taking my child out |
|  | A2 | Make plans to ensure my child’s safety at school or daycare |
|  | A3 | Make plans to ensure my child’s safety when they are with relatives, friends, or a babysitter |
|  | A4 | Plan to attend social activities involving food (e.g. parties) with others |
|  | A5 | Teach others about my child’s food allergy |
|  | A6 | Manage my child’s environment to prevent accidental exposure to allergens |
| B: Allergic reaction | B1 | Recognize my child’s allergic reactions |
|  | B2 | Treat my child with appropriate non-pharmacological measures if they had an allergic reaction, such as immediately discontinuing the consumption of the food that triggered the allergy |
|  | B3 | Treat my child with appropriate pharmacological measures if they had an allergic reaction, such as taking cetirizine as prescribed by the physician. |
|  | B4 | Observe and clarify the progression of my child's allergic reaction |
| C: Food allergy identification | C1 | Read food labels for information |
|  | C2 | Identify potential food cross-contamination (where previously safe foods become contaminated with allergenic substances). |
|  | C3 | Prepare homemade meals |
| D: Seeking information about food allergy |  | Obtain information about my child’s food allergies from the following sources: |
|  | D1 | Doctors or nurses at community health service centers |
|  | D2 | Paediatrician or allergy specialist at the hospital |
|  | D3 | Food retailers (e.g. supermarkets, food outlets) |
|  | D4 | Websites |
|  | D5 | Books |
|  | D6 | Other parents of children with food allergies |
| E: Managing social activities around food allergy | E1 | Prepare before going to restaurants |
|  | E2 | Dine at restaurants (without triggering food allergies in children) |
|  | E3 | Make plans for vacations within the country |
|  | E4 | Vacation within the country (without triggering food allergies in children) |
|  | E5 | Make plans for vacations abroad |
|  | E6 | Vacation abroad (without triggering food allergies in children) |

**Supplementary Table S3** **Factor loadings of the initial EFA for CAR-FASE-P (version 3).**

| Item Number | Factor 1 | Factor 2 | Factor 3 | Factor 4 |
| --- | --- | --- | --- | --- |
| A1 | 0.079 | 0.288 | 0.019 | **0.799** |
| A2 | 0.152 | 0.206 | 0.185 | **0.772** |
| A3 | 0.102 | 0.202 | 0.027 | **0.711** |
| A4 | -0.070 | **0.578** | 0.260 | 0.346 |
| A5 | 0.126 | 0.120 | -0.005 | **0.654** |
| A6 | **0.451** | 0.018 | 0.125 | **0.631** |
| B1 | **0.723** | 0.086 | 0.330 | 0.118 |
| B2 | **0.820** | 0.073 | 0.204 | 0.1751 |
| B3 | **0.718** | 0.077 | -0.040 | 0.130 |
| B4 | **0.809** | 0.255 | 0.087 | 0.137 |
| C1 | **0.765** | 0.022 | 0.136 | 0.266 |
| C2 | **0.579** | 0.169 | 0.114 | 0.182 |
| C3 | **0.778** | 0.202 | 0.105 | -0.059 |
| D1 | -0.016 | 0.217 | **0.660** | 0.135 |
| D2 | **0.644** | 0.443 | 0.151 | 0.006 |
| D3 | 0.178 | 0.069 | **0.783** | -0.060 |
| D4 | 0.220 | 0.181 | **0.795** | 0.000 |
| D5 | 0.076 | 0.303 | **0.792** | 0.248 |
| D6 | 0.389 | 0.155 | **0.744** | 0.051 |
| E1 | **0.501** | **0.579** | 0.077 | 0.021 |
| E2 | 0.097 | **0.712** | 0.143 | 0.256 |
| E3 | 0.165 | **0.773** | 0.217 | 0.273 |
| E4 | 0.315 | **0.796** | 0.180 | 0.094 |
| E5 | 0.204 | **0.815** | 0.152 | 0.201 |
| E6 | 0.169 | **0.810** | 0.166 | 0.114 |
| Cumulative Variance Contribution Rate (%) | 37.157 | 48.521 | 57.844 | 64.990 |

**
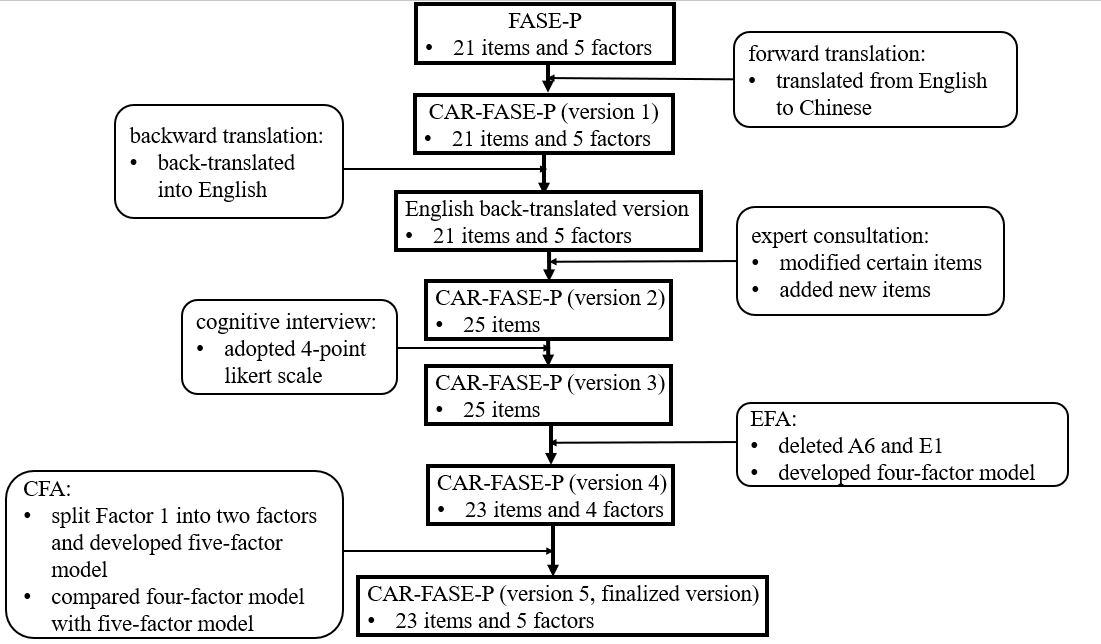
**

**Supplementary Figure S1 The generation process for the different versions of the** **CAR-FASE-P.**

**Supplementary Table S4 The dimensions and items of the finalized CAR-FASE-P in English.**

| Dimension | Item |
| --- | --- |
| Precaution and prevention of an allergic reaction | Be well-prepared before taking my child out |
|  | Make plans to ensure my child’s safety at school or daycare |
|  | Make plans to ensure my child’s safety when they are with relatives, friends, or a babysitter |
|  | Teach others about my child’s food allergy |
| Allergic reaction | Recognize my child’s allergic reactions |
|  | Treat my child with appropriate non-pharmacological measures if they had an allergic reaction, such as immediately discontinuing the consumption of the food that triggered the allergy |
|  | Treat my child with appropriate pharmacological measures if they had an allergic reaction, such as taking cetirizine as prescribed by the physician. |
|  | Observe and clarify the progression of my child's allergic reaction |
| Food allergy identification | Read food labels for information |
|  | Identify potential food cross-contamination (where previously safe foods become contaminated with allergenic substances). |
|  | Prepare homemade meals |
| Seeking information about food allergy | Obtain information about my child’s food allergies from the following sources: |
|  | Doctors or nurses at community health service centers |
|  | Paediatrician or allergy specialist at the hospital |
|  | Food retailers (e.g. supermarkets, food outlets) |
|  | Websites |
|  | Books |
|  | Other parents of children with food allergies |
| Managing social activities around food allergy | Plan to attend social activities involving food (e.g. parties) with others |
|  | Dine at restaurants (without triggering food allergies in children) |
|  | Make plans for vacations within the country |
|  | Vacation within the country (without triggering food allergies in children) |
|  | Make plans for vacations abroad |
|  | Vacation abroad (without triggering food allergies in children) |

**Supplementary** **Table S5 Sensitive analysis: model fit indices of the CFA for parents of FA children aged < 1 Year (n=243).**

|  | χ^2^/df | RMSEA (95% CI) | CFI | TLI | WRMR |
| --- | --- | --- | --- | --- | --- |
| Five-factor model | 1.148 | 0.025(0.000, 0.038) | 0.999 | 0.999 | 0.886 |
| Cut-off values | < 3 | ≤ 0.06 | ≥ 0.97 | ≥ 0.97 | ≤0.95 |

**Supplementary Table S6 Sensitive analysis: Factor loadings of the** **initial EFA based on random number table sampling (n=150).**

| Item Number | Factor 1 | Factor 2 | Factor 3 | Factor 4 | |
| --- | --- | --- | --- | --- | --- |
| A1 | 0.049 | 0.328 | 0.108 | | **0.770** |
| A2 | 0.213 | 0.109 | 0.366 | | **0.732** |
| A3 | 0.111 | 0.180 | -0.017 | | **0.766** |
| A4 | -0.008 | **0.534** | 0.324 | | 0.355 |
| A5 | 0.311 | 0.291 | -0.063 | | **0.347** |
| A6 | **0.536** | 0.225 | 0.083 | | **0.409** |
| B1 | **0.726** | 0.120 | 0.176 | | 0.215 |
| B2 | **0.773** | 0.205 | 0.180 | | 0.171 |
| B3 | **0.798** | 0.086 | 0.020 | | 0.142 |
| B4 | **0.767** | 0.350 | 0.060 | | 0.174 |
| C1 | **0.853** | 0.112 | 0.065 | | 0.132 |
| C2 | **0.565** | 0.290 | 0.259 | | -0.064 |
| C3 | **0.692** | 0.313 | 0.151 | | -0.101 |
| D1 | -0.024 | 0.072 | **0.835** | | 0.067 |
| D2 | 0.388 | 0.126 | **0.402** | | 0.000 |
| D3 | 0.069 | 0.040 | **0.811** | | -0.029 |
| D4 | 0.162 | 0.256 | **0.647** | | 0.423 |
| D5 | 0.181 | 0.276 | **0.647** | | 0.423 |
| D6 | 0.371 | 0.155 | **0.581** | | 0.011 |
| E1 | **0.499** | **0.626** | 0.037 | | 0.081 |
| E2 | 0.198 | **0.751** | -0.033 | | 0.277 |
| E3 | 0.305 | **0.818** | 0.191 | | 0.177 |
| E4 | 0.280 | **0.829** | 0.143 | | 0.146 |
| E5 | 0.212 | **0.761** | 0.245 | | 0.155 |
| E6 | 0.217 | **0.785** | 0.235 | | 0.196 |
| Cumulative Variance Contribution Rate (%) | 20.896 | 39.498 | 52.436 | | 63.428 |

**Supplementary Table S7 Sensitive analysis: factor loadings of the second EFA based on random number table sampling (n=150).**

| Item Number | Factor 1 | Factor 2 | Factor 3 | Factor 4 | |
| --- | --- | --- | --- | --- | --- |
| A1 | 0.062 | 0.299 | 0.099 | | **0.803** |
| A2 | 0.221 | 0.132 | 0.348 | | **0.713** |
| A3 | 0.109 | 0.154 | -0.021 | | **0.789** |
| A4 | 0.013 | **0.511** | 0.314 | | 0.390 |
| A5 | 0.319 | 0.311 | -0.083 | | **0.327** |
| B1 | **0.728** | 0.150 | 0.156 | | 0.175 |
| B2 | **0.766** | 0.222 | 0.170 | | 0.132 |
| B3 | **0.800** | 0.067 | 0.006 | | 0.151 |
| B4 | **0.775** | 0.333 | 0.043 | | 0.182 |
| C1 | **0.855** | 0.104 | 0.047 | | 0.130 |
| C2 | **0.589** | 0.230 | 0.245 | | 0.009 |
| C3 | **0.714** | 0.237 | 0.139 | | -0.019 |
| D1 | -0.019 | 0.116 | **0.835** | | 0.027 |
| D2 | **0.409** | 0.170 | 0.374 | | -0.030 |
| D3 | 0.081 | 0.026 | **0.815** | | -0.005 |
| D4 | 0.182 | 0.195 | **0.604** | | 0.356 |
| D5 | 0.193 | 0.284 | **0.638** | | 0.421 |
| D6 | 0.385 | 0.134 | **0.575** | | 0.039 |
| E2 | 0.217 | **0.723** | -0.047 | | 0.310 |
| E3 | 0.330 | **0.805** | 0.172 | | 0.199 |
| E4 | 0.294 | **0.820** | 0.132 | | 0.153 |
| E5 | 0.221 | **0.823** | 0.230 | | 0.088 |
| E6 | 0.224 | **0.829** | 0.223 | | 0.145 |
| Cumulative Variance Contribution Rate (%) | 21.069 | 39.256 | 52.866 | | 64.400 |

**Supplementary Table S8 Sensitive analysis: model fit indices of the CFA in the randomly generated sample (n=222).**

|  | χ^2^/df | RMSEA (95% CI) | CFI | TLI | WRMR |
| --- | --- | --- | --- | --- | --- |
| Five-factor model | 1.103 | 0.022 (0.000, 0.037) | 0.999 | 0.999 | 0.869 |
| Cut-off values | < 3 | ≤ 0.06 | ≥ 0.97 | ≥ 0.97 | ≤0.95 |
